# Laparoscopic Small Bowel Length Measurement in Bariatric Surgery Using a Hand-Over-Hand Technique with Marked Graspers: an Ex Vivo Experiment

**DOI:** 10.1007/s11695-022-05918-z

**Published:** 2022-02-24

**Authors:** Nienke Slagter, Mette van Wilsum, Loek J. M. de Heide, Ewoud H. Jutte, Mirjam A. Kaijser, Stefan L. Damen, André P. van Beek, Marloes Emous

**Affiliations:** 1 Center for Obesity Northern Netherlands (CON), Medical Center Leeuwarden, Henri Dunantweg 2, 8934 AD Leeuwarden, the Netherlands; 2 University Medical Center Groningen, University of Groningen, Hanzeplein 1, 9713 GZ Groningen, the Netherlands; 3 Department of Endocrinology, University of Groningen, University Medical Center Groningen, Hanzeplein 1, 9713 GZ Groningen, the Netherlands

**Keywords:** Bariatric surgery, Limb length, Small bowel length, Laparoscopic bowel length measurement

## Abstract

**Introduction:**

Tailoring limb length in bariatric surgery is a subject of many studies. To acquire the optimal limb length, accurate measurement of the small bowel length is essential.

**Objective:**

To assess the intra- and inter-individual variability of laparoscopic bowel length measurement using a hand-over-hand technique with marked graspers.

**Method:**

Four bariatric surgeons and four surgical residents performed measurements on cadaver porcine intestine in a laparoscopic box using marked graspers. Each participant performed 10 times a measurement of three different lengths: 150, 180, and 210 cm. Acceptable percentage deviation from the goal lengths was defined as less than 10%, while unacceptable deviations were defined as more than 15%.

**Results:**

The bariatric surgeons measured the 150-, 180-, and 210-cm tasks with 4% (*CI* 0.4, 9), − 6% (*CI* − 11, − 0.8), and 1% (*CI* − 4, 6) deviation, respectively. In total, the bariatric surgeons estimated 58 out of 119 times (49%) between the margins of 10% deviation and 36 times (30%) outside the 15% margin. Considerable inter-individual differences were found between the surgeons. The surgical residents underestimated the tasks with 12% (*CI* − 18, − 6), 16% (*CI* − 19, − 13), and 18% (*CI* − 22, − 13), respectively.

**Conclusion:**

Bariatric surgeons estimated bowel length with on average less than 10% deviation. However, this still resulted in 30% of the measurements with more than 15% deviation. There were considerable inter-individual differences between the surgeons and residents structurally underestimated the bowel length. Ascertainment of measurement accuracy and adequate training is essential for bariatric procedures in which limb length is of importance.

**Graphical abstract:**

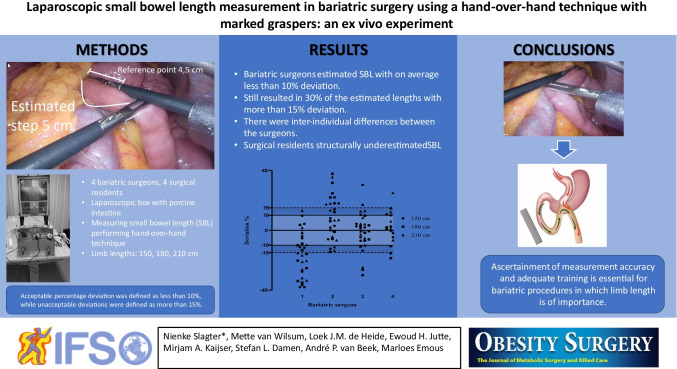

## Introduction

The Roux-en-Y gastric bypass (RYGB), the one anastomosis gastric bypass (OAGB), and single anastomosis duodeno-ileal bypass with sleeve gastrectomy (SADI-S) are effective and often performed bariatric procedures with comparable outcomes of weight loss and nutritional deficiencies [1–3]. The optimal lengths of the alimentary limb and biliopancreatic (BP) limb remain a topic of discussion. The aim of an optimal limb length is to achieve ideal results in terms of weight loss while minimizing the chance of nutritional deficiencies [4–6]. In RYGB, a longer alimentary limb seems to have no influence on weight loss outcomes, whereas a longer BP-limb results in more weight loss overall [6–8].

In the discussion about the optimal limb length, the accuracy and precision of limb length measurement are essential. Inadequate measurement results in over- or under-estimation of a bowel segment. This may have clinical consequences in terms of weight loss, rates of malnutrition, and vitamin deficiencies. In gastric bypass surgery different techniques are performed to measure the small bowel length [9]. Some surgeons use a rope or ruler to measure the segments of the small bowel [10]. Others use graspers or other laparoscopic instruments as a reference tool to stepwise estimate the length of the small bowel [11]. Regardless of the performed measurement method, laparoscopic small bowel measurement remains challenging due to the influence of limited range of motion, the high flexibility of the bowel structure, and the two-dimensional imaging of a three-dimensional bowel [12]. There is limited literature investigating the accuracy of laparoscopic small bowel measurement performed in bariatric surgery. The aim of this study was to assess the intra- and the inter-individual variability of laparoscopic small bowel length measurement using a hand-over-hand technique with marked graspers in an ex vivo experiment.

## Method

### Participants

This is a single-center ex vivo experiment including all four bariatric surgeons and four surgical residents of a non-academic teaching hospital in the northern Netherlands. Baseline data were collected of all participants including, age, gender, laparoscopic experience, and bariatric experience. For cadaver studies, no ethical approval is required in the Netherlands. The intestine of the porcine cadavers was supplied by a registered slaughterhouse and the use of animal by-products was approved by the Netherlands Food and Consumer Product Safety Authority.

### Laparoscopic Simulation

**Fig. 1:**
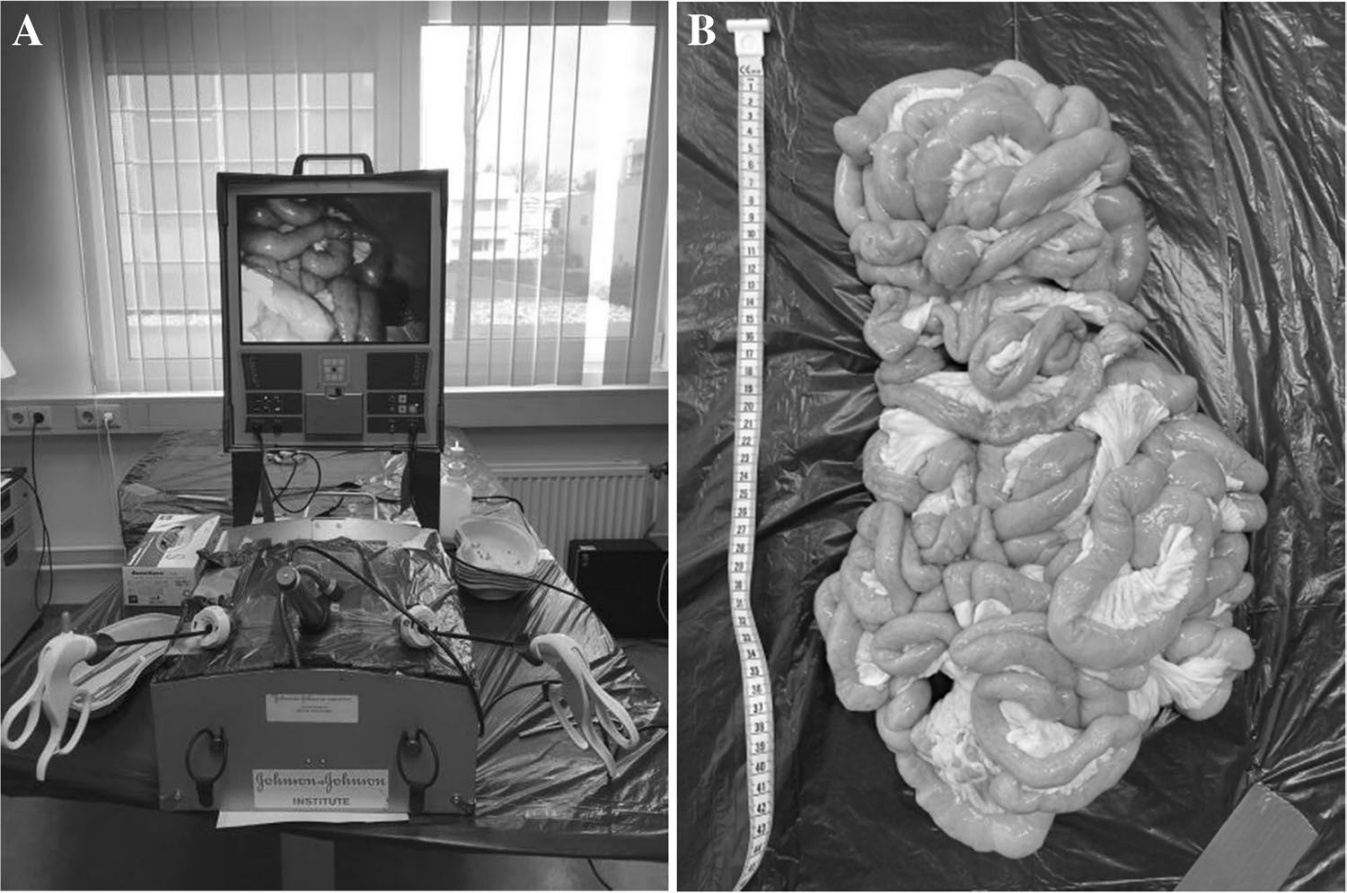
Laparoscopic set up. **A** Laparoscopic box trainer **B** small bowel of the porcine

A laparoscopic box training system (Lapstar model 2, Camtronics B.V. Son, Netherlands) with a fixed camera was used to simulate the laparoscopic abdominal surgery. The total small bowel of a porcine cadaver was placed in the laparoscopic box (Fig. 1). Participants were instructed to perform the stepwise hand-over-hand technique using two metallic graspers to pass the small bowel in estimated steps of 5 cm (video 1). The length of the antimesenteric border is measured with the bowel in normal position and without applying traction. In our bariatric center, the hand-over-hand measurement technique is the laparoscopic measurement method performed in daily practice. Comparable with the used graspers in bariatric surgery, one of the instruments had a 4.5-cm marker as a reference point.

### Laparoscopic Bowel Measurement

Each participant completed three rounds of 10 measurements on different days, estimating randomly assigned lengths of 150, 180, and 210 cm. The start and endpoints of the measurements were marked with color-coded sutures. Two observers, blinded to the assigned tasks, each measured the actual length of the marked segments outside the laparoscopic box trailer using a tape measure*.* These measurements were performed immediately after the laparoscopic measurements were completed, to minimize the time difference between the laparoscopic and tape measurements. The bowel was measured with the same technique as the laparoscopic measurements: at the antimesenteric border with the bowel in normal position and without applying traction. The mean of the two tape measure lengths was used as actual measured length. The participants did not receive any feedback on their results until after the experiment to eliminate any influence of learning.

### Statistical Analysis

**Fig. 2:**
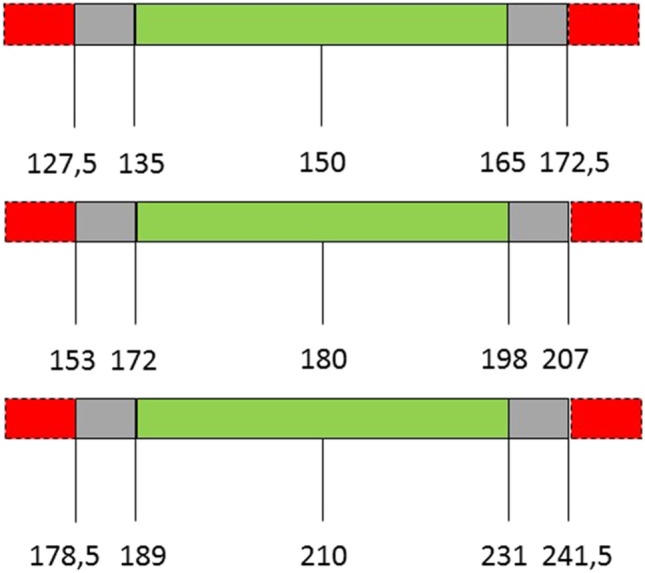
The acceptable cut-off value of 10% and the unacceptable cut-off point of 15% for the different measured limb lengths

The individual scores of the participants were presented as mean ± standard deviation. The percentage deviation from the goal lengths were analyzed by a mixed model for repeated measures analyses, correcting for the dependency of the repeated measurements. The percentage deviation measured by the bariatric surgeons was compared to the surgical residents. Individual percentage deviation of the bariatric surgeons was compared for each measured task. Acceptable variation in small bowel length measurement was defined as less than 10% deviation from the actual bowel length. Unacceptable variation was defined as more than 15% deviation from the actual bowel length (Fig. 2). Statistical analyses were assessed in SAS/STAT® software.

## Results

One measured value was excluded due to an incorrect measurement. Three of the bariatric surgeons have performed more than 2000 laparoscopic bariatric surgical procedures during their career and one of the bariatric surgeons performed more than 500 bariatric surgical procedures (Table 5, appendix). As shown in Table 1, the mean measured lengths of the 150-cm task by the four bariatric surgeons individually were 131 ± 25, 158 ± 17, 156 ± 19, and 158 ± 10 cm, respectively. As a group, the surgeons estimated 22 out of 39 times (56%) between the lower and upper margins of 10% deviation and 7 times (18%) outside the 15% margin. Mean measured values on the 180 cm distance were 145 ± 24, 205 ± 29, 168 ± 23, and 172 ± 21 cm, respectively. The estimated lengths on the 180-cm task were 18 out of 40 times between the lower and upper margins (45%) and 16 times (40%) outside the 15% margin. On the 210-cm task, the surgeons measured 176 ± 31, 223 ± 32, 210 ± 24, and 221 ± 24 cm. The measurements were 18 out of 40 times between the 10% margins (45%) and 13 times (33%) outside the 15% margin. As shown in Table 2, the surgical residents measured a mean length of 137 ± 31 cm on the 150-cm task and 159 ± 32 cm on the 180-cm task. Mean measured length on the 210-cm task was 183 ± 40 cm. In total, the residents estimated the lengths 25 out of 120 times between the 10% margin (21%).

**Table 1:** Individual measured values of the bariatric surgeons

	Bariatric surgeon 1	Bariatric surgeon 2	Bariatric surgeon 3	Bariatric surgeon 4	Total
150 cm, *n*	10	9	10	10	39
Measured, cm	131 ± 25	158 ± 17	156 ± 19	158 ± 10	151 ± 21
Deviation, cm	− 19 ± 25	8 ± 17	6 ± 19	8 ± 10	0.7 ± 21
Deviation, %	− 12 ± 17	5 ± 11	4 ± 13	5 ± 7	0.5 ± 15
Absolute deviation, %	15 ± 14	9 ± 8	9 ± 9	7 ± 5	10 ± 10
Between margins 10%	5 of 10	5 of 9	6 of 10	6 of 10	22 of 39
Outside 15% margins	3 of 10	2 of 9	2 of 10	0 of 10	7 of 39
180 cm, *n*	10	10	10	10	40
Measured, cm	145 ± 24	205 ± 29	168 ± 23	172 ± 21	172 ± 32
Deviation, cm	− 35 ± 24	25 ± 29	− 12 ± 23	− 8 ± 21	− 8 ± 32
Deviation, %	− 20 ± 13	14 ± 16	− 7 ± 13	− 5 ± 11	− 4 ± 18
Absolute deviation, %	20 ± 12	17 ± 12	10 ± 10	10 ± 7	14 ± 11
Between margins 10%	3 of 10	3 of 10	7 of 10	5 of 10	18 of 40
Outside 15% margins	6 of 10	5 of 10	2 of 10	3 of 10	16 of 40
210 cm, *n*	10	10	10	10	40
Measured, cm	176 ± 31	223 ± 32	210 ± 24	221 ± 24	208 ± 33
Deviation, cm	− 34 ± 31	13 ± 32	− 0.3 ± 24	11 ± 24	− 3 ± 33
Deviation, %	− 16 ± 15	6 ± 15	− 0.2 ± 11	5 ± 11	− 1 ± 16
Absolute deviation, %	19 ± 10	13 ± 9	9 ± 7	10 ± 7	13 ± 9
Between margins 10%	2 of 10	4 of 10	7 of 10	5 of 10	18 of 40
Outside 15% margins	7 of 10	3 of 10	1 of 10	2 of 10	13 of 40

Values are mean ± standard deviation

**Table 2:** Individual measured values of the residents

	Resident 1	Resident 2	Resident 3	Resident 4	Total
150 cm, *n*	10	10	10	10	40
Measured, cm	108 ± 12	172 ± 28	132 ± 24	138 ± 18	137 ± 31
Deviation, cm	− 42 ± 12	22 ± 28	− 18 ± 24	− 12 ± 18	− 13 ± 31
Deviation, %	− 28 ± 8	14 ± 19	− 12 ± 16	− 8 ± 12	− 8 ± 21
Absolute deviation, %	28 ± 8	18 ± 14	17 ± 10	12 ± 8	19 ± 12
Between margins 10%	0 of 10	3 of 10	3 of 10	5 of 10	11 of 40
Outside 15% margins	9 of 10	6 of 10	5 of 10	3 of 10	23 of 40
180 cm, *n*	10	10	10	10	40
Measured, cm	130 ± 15	202 ± 28	149 ± 9	154 ± 9	159 ± 32
Deviation, cm	− 50 ± 15	22 ± 28	− 31 ± 16	− 26 ± 9	− 21 ± 32
Deviation, %	− 28 ± 8	12 ± 16	− 17 ± 9	− 14 ± 5	− 12 ± 18
Absolute deviation, %	28 ± 8	15 ± 12	18 ± 9	14 ± 5	19 ± 10
Between margins 10%	0 of 10	3 of 10	1 of 10	2 of 10	6 of 40
Outside 15% margins	10 of 10	4 of 10	6 of 10	4 of 10	24 of 40
210 cm, *n*	10	10	10	10	40
Measured, cm	148 ± 20	229 ± 28	180 ± 39	175 ± 18	183 ± 40
Deviation, cm	− 62 ± 20	19 ± 28	− 30 ± 39	− 35 ± 18	− 27 ± 40
Deviation, %	− 29 ± 9	9 ± 13	− 14 ± 19	− 17 ± 8	− 13 ± 19
Absolute deviation, %	29 ± 9	12 ± 10	20 ± 12	17 ± 8	20 ± 11
Between margins 10%	0 of 10	4 of 10	2 of 10	2 of 10	8 of 40
Outside 15% margins	10 of 10	4 of 10	6 of 10	7 of 10	27 of 40

Values are mean ± standard deviation

Deviation percentage of the goal length was compared between the surgeons and residents for each task separately (Table 3). The bariatric surgeons overestimated the 150-cm and 210-cm tasks with 4% (*CI* 0.4, 9) and 1% (*CI* − 4, 6), respectively. The 180-cm goal length was underestimated by the surgeons with 6% (*CI* − 11, − 0.8). The surgical residents underestimated the lengths of the 150-, 180-, and 210-cm tasks with 12 (*CI* − 18, − 6), 16 (*CI* − 19, − 13), and 18% (*CI* − 22, − 13), respectively. There was a significant difference between the surgeons and residents on all three measured tasks.

**Table 3:** Repeated measures analyses for the difference between surgeons and residents

	Estimate	*P*-value	95% *CI*	
			Lower	Upper
150 cm, *n* = 79				
Bariatric surgeons	4	0.03	0.4	9
Residents	− 12	0.0005	− 18	− 6
Differences				
Surgeons vs residents	16	< 0.001	9	23
180 cm, *n* = 80				
Bariatric surgeons	− 6	0.03	− 11	− 1
Residents	− 16	< 0.001	− 19	− 13
Differences				
Surgeons vs residents	10	0.003	4	16
210 cm, *n* = 80				
Bariatric surgeons	1	0.56	− 4	6
Residents	− 18	< 0.001	− 22	− 13
Differences				
Surgeons vs residents	19	< 0.001	12	26

Dependent variable: percentage deviation (%). Estimates of repeated measures analyses were determined for the group of bariatric surgeons and surgical residents. Estimates were compared to analyze the differences between bariatric surgeons and surgical residents. *CI*, confidence interval

**Fig. 3:**
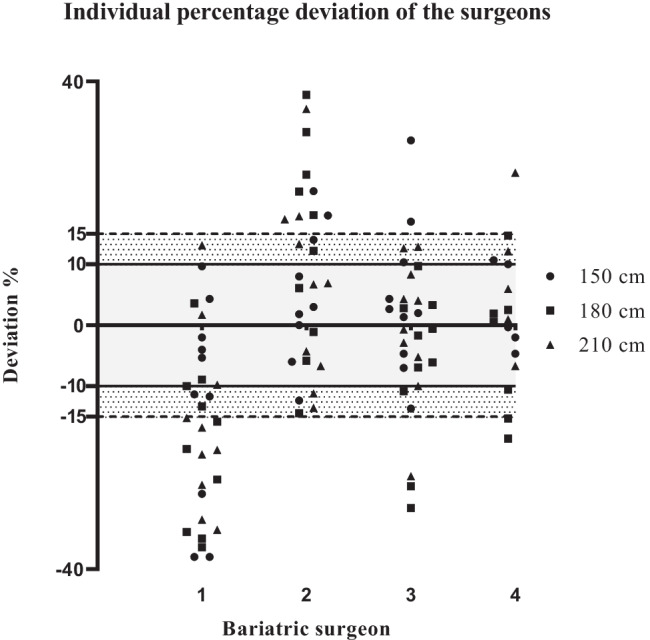
Percentage deviation on all three tasks measured by the bariatric surgeons

The individual deviation percentage and the inter-individual difference between the surgeons are shown in Table 4 and Fig. 3. Bariatric surgeon 1 underestimated the lengths of all three task, with 12 (*CI* − 24, − 0.4), 20 (*CI* − 29, − 10), and 16% (*CI* − 26, − 6), respectively. There were significant differences between surgeon 1 and the other three surgeons on all three measured tasks. On the 150-cm task, surgeon 2, 3, and 4 overestimated the length showing no difference between each other. On the 180-cm goal length, an overestimation was seen in surgeon 2, which significantly differed compared to the other surgeons. Surgeon 2, 3, and 4 showed no difference on the 210-cm task.

**Table 4:** Repeated measures analyses and inter-individual difference of the bariatric surgeons

	Estimate	*P*-value	95% *CI*	
			Lower	Upper
150 cm, *n* = 39				
Bariatric surgeon 1	− 12	0.04	− 24	− 0.4
Bariatric surgeon 2	5^1^	0.19	− 3	14
Bariatric surgeon 3	4^1^	0.31	− 5	13
Bariatric surgeon 4	5^1^	0.045	0.1	10
180 cm, *n* = 40				
Bariatric surgeon 1	− 20	0.001	− 29	− 10
Bariatric surgeon 2	14^1^	0.03	2	25
Bariatric surgeon 3	− 7^12^	0.13	− 16	2
Bariatric surgeon 4	− 5^12^	0.23	− 13	4
210 cm, *n* = 40				
Bariatric surgeon 1	− 16	0.007	− 26	− 6
Bariatric surgeon 2	6^1^	0.24	− 5	17
Bariatric surgeon 3	− 0.2^1^	0.97	− 8	8
Bariatric surgeon 4	5^1^	0.18	− 3	13

Dependent variable: percentage deviation (%). Estimates of repeated measures analyses were determined for each bariatric surgeon individually. *CI*, confidence interval

^1^Significant difference compared to bariatric surgeon 1

^2^Significant difference compared to bariatric surgeon 2

## Discussion

This study shows that experienced bariatric surgeons estimate laparoscopic small bowel length with on average less than 10% deviation from the goal lengths. However, this still resulted in 51% of the estimated bowel lengths with more than 10% deviation and 30% with more than 15% deviation from the goal lengths. There were considerable inter-individual differences between the bariatric surgeons. Furthermore, surgical residents inaccurately estimated the small bowel length by structurally underestimating the limb lengths.

Acceptable variation in bowel length measurement was defined as less than 10% deviation from the goal limb length. Due to lack of literature, it is unknown which percentage deviation is still acceptable without causing clinical consequences in terms of weight loss and nutritional deficiencies. A deviation of 10% was used as acceptable cut-off value, because a higher deviation would result in an overlap of the different limb lengths used in bariatric surgery (Fig. 2). A more liberal margin still resulted in 30% of measurements outside 15% deviation. These percentages outside the margins can partially be explained by the inter-individual difference between the bariatric surgeons, as one of the bariatric surgeons structurally underestimated the small bowel length with more than 10% deviation. With regard to the structural underestimation, this is probably due to repeatedly estimating the steps too small. Nevertheless, the other three surgeons estimated 22% of the bowel segments with more than 15% deviation. With the hand-over-hand technique, the estimated steps can easily be affected by multiple factors such as the flexible structure of the small bowel with limited stretch due to the mesentery, two-dimensional imaging of a three-dimensional bowel, counting error, or error of judgement, which all can result in outlying measurements. In daily practice, these deviations can result in bariatric patients with considerable difference between the goal and actual limb length. Nevertheless, the actual clinical consequences of these variations remain as yet unknown.

Comparable studies show deviations which are considerably higher compared to our results. The study of Gazer et al. assessed the reliability of laparoscopic bowel length measurement in 14 surgeons using an in vivo porcine model. They found that measured lengths were 36% shorter than the actual length, concluding that the assessment during laparoscopy was inaccurate [13]. However, the measurements were not performed by bariatric surgeons, which may explain the different outcomes, as general surgeons perform laparoscopic small bowel measurements to a much lesser extent than bariatric surgeons. Furthermore, they used non-marked laparoscopic graspers as a reference tool. A study of Isreb et al. investigated the effect of marking the graspers on measurement precision using a piece of string and a laparoscopic box trainer [11]. Greater accuracy was found for the measurements performed with marked instruments. Furthermore, a study of Lusseden et al. evaluated the accuracy of stepwise laparoscopic small bowel measurement in residents and attendings using 500-cm porcine intestine in a laparoscopic box trainer [14]. Both residents and attendings measured averaged 24 cm away from the 100-cm goal, concluding there is a wide variability in both residents and attendings. They concluded that both groups should be educated to measure small bowel length more accurately. However, the study included no bariatric surgeons, and the participants performed several different step sizes to measure the bowel length. Another study compared the accuracy and precision of laparoscopic measurement with and without the aid of a measuring tool, using a laparoscopic box trainer and a rope [15]. The surgical residents underestimated with a mean of 128 ± 42 cm on the 150-cm goal length, comparable with the results of the residents in our experiment. Nevertheless, it is unknown if estimation with a rope is adequate with small bowel measurement, as it excludes relevant factors like the sensation and flexible structure of the intestinal tissue and the limited stretch of the mesentery.

Considering the inter-individual differences between the bariatric surgeons, all four surgeons had learned the hand-over-hand measurement technique in vivo from more experienced bariatric surgeons. Those experienced bariatric surgeons provided the surgeons feedback and decided whether they accurately estimated the limb lengths. Possible different learning methods, effects, and subjective assessments may have contributed to differences in results between the bariatric surgeons.

To our knowledge, this is the first study investigating the intra- and the inter-individual variability of stepwise hand-over-hand laparoscopic bowel measurement in bariatric surgeons using a representative ex vivo model. As this stepwise measurement technique with marked graspers is used in a wide number of bariatric centers over the world, this is an important first step in evaluating this method in gastric bypass surgery [16]. In our bariatric center, this technique is performed in daily practice. Based on the results of the study, we continue using this measurement technique in gastric bypass surgery. The bariatric surgeon who structurally underestimated the lengths has adapted his measurement steps when performing the hand-over-hand measurement technique. As optimal limb length in bariatric surgery is a subject of many studies, differences between goal and actual limb length and differences among surgeons have possible consequences for studies investigating limb lengths. The results of this study emphasize that studies investigating limb lengths should incorporate results on the intra- and inter-individual variability of bowel length measurement in their center. It highlights the need for research to optimize this laparoscopic measurement technique and to evaluate different bowel length measurement techniques in bariatric surgery. Furthermore, the inaccurate estimation of the limb lengths by the surgical residents and the inter-individual differences between the bariatric surgeons both emphasize the need for a standardized learning method.

Limitations of this study were the use of an ex vivo model with devascularized porcine intestine, which caused a different sensation of the tissue and excluded the relevant factor of peristaltic bowel movements. The quality of the porcine intestine decreased during the day, which may have affected the measurements at the end of the day. An in vivo model would be preferable, but with the ex vivo model, it was possible to compare the estimated bowel lengths with the actual goal lengths, without having to perform animal experiments. Porcine intestine has several similarities with the human bowel, making it an appropriate model for this experiment [17]. The flexibility and sticky characteristics of the devascularized porcine intestine may have affected the tape measures determining the actual limb lengths; therefore, these measurements can also deviate from the actual limb lengths. Tape measures were performed twice to minimize this deviation. This study has a limited sample size with only four bariatric surgeons. However, this might be an adequate reflection of several bariatric centers, employing approximately four bariatric surgeons.

In conclusion, bariatric surgeons performed laparoscopic small bowel length measurements with an average less than 10% deviation from the goal lengths. However, this still resulted in 30% of the estimated lengths with more than 15% deviation. There were considerable inter-individual differences between the bariatric surgeons. Surgical residents structurally underestimated the limb lengths. Ascertainment of measurement accuracy and sufficient training of bariatric surgeons is essential for bariatric surgeries in which the limb length is of importance.

## Appendix

Tables 5 and 6

**Table 5:** Participant demographics

	Sex	Age	Years of experience/residency	Lap experience	Bariatric experience	Lap bowel measurement
Bariatric surgeon 1	Male	46	11	> 3000	> 2000	> 1500
Bariatric surgeon 2	Male	43	11	> 3000	> 2000	> 1500
Bariatric surgeon 3	Female	45	10	> 3000	> 2000	> 1500
Bariatric surgeon 4	Female	36	5	> 1000	> 500	> 500
Resident 1	Male	34	3	50–100	-	10–20
Resident 2	Male	32	1	1–10	-	1–10
Resident 3	Male	30	1	10–50	-	0
Resident 4	Female	32	2	1–10	-	1–10

Lap experience, laparoscopic experience expressed as number of laparoscopic surgeries; lap bowel measurement, laparoscopic bowel measurement expressed as number of performed laparoscopic bowel measurements

**Table 6:** Inter-individual difference between bariatric surgeons

	Bariatric surgeon 2		Bariatric surgeon 3		Bariatric surgeon 4	
	Estimate	*P*-value	Estimate	*P*-value	Estimate	*P*-value
Bariatric surgeon 1						
150 cm	− 18	0.01	− 17	0.02	− 18	0.01
180 cm	− 33	0.0001	− 13	0.04	− 15	0.01
210 cm	− 22	0.004	− 16	0.02	− 21	0.002
Bariatric surgeon 2						
150 cm			1	0.84	0.3	0.95
180 cm			20	0.006	18	0.01
210 cm			6	0.31	1	0.87
Bariatric surgeon 3						
150 cm					− 1	0.85
180 cm					− 2	0.71
210 cm					− 5	0.31

Dependent variable: percentage deviation (%). Estimates of each bariatric surgeons individually were compared to analyze the inter-individual differences
